# *Ab initio* kinetics predictions for the role of pre-reaction complexes in hydrogen abstraction from 2-butanone by OH radicals†

**DOI:** 10.1039/d0ra05332e

**Published:** 2020-09-08

**Authors:** Yi Gao, Yang Zhao, Qingbao Guan, Fuke Wang

**Affiliations:** Center for Combustion Energy, Key Laboratory for Thermal Science and Power Engineering of MOE, Tsinghua University Beijing 100084 China y-g18@mails.tsinghua.edu.cn; Soft Materials, Institute of Materials Research and Engineering, Agency for Science, Technology and Research (A*STAR) 2 Fusionopolis Way, #08-03 Innovis 138634 Singapore; State Key Laboratory for Modification of Chemical Fibers and Polymer Materials, International Joint Laboratory for Advanced Fiber and Low-dimension Materials, College of Materials Science and Engineering, Donghua University Shanghai 201620 P. R. China

## Abstract

The existence of pre- and post-reaction complexes has been proposed to influence hydrogen abstraction reaction kinetics, but the significance still remains controversial. A theoretical study is presented to discuss the effects of complexes on hydrogen abstraction from 2-butanone by OH radicals based on the detailed PESs at the DLPNO-CCSD(T)/aug-cc-pVTZ//M06-2x-D3/may-cc-pVTZ level with five pre-reaction complexes at the entrance of the channels and four post-reaction complexes at the exit. The hydrogen bond interactions, steric effects, and contributions to the bonding orbital of the OH radical species and 2-butanone species in the complex structures were visualized and investigated by wavefunction analyses. Three kinds of mechanisms—the general bimolecular reaction, the reaction with the complexes considered, and the well-skipping reaction—were compared based on high-pressure-limit rate constants, predicted branching ratios, and fractional populations of reactants and products in the temperature range of 250–2000 K. The existence of complexes was proved to be crucial in the kinetics and mechanisms of the hydrogen abstraction from 2-butanone molecules by OH radicals.

## Abstract 2

Schematic diagram of the geometry and corresponding weak interactions in the complex between two reactant monomers.

## Graphical abstract

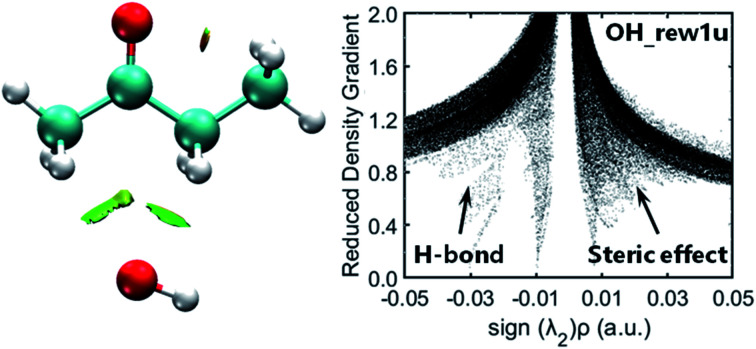

## 1. Introduction

Oxygenated fuels and fuel additives are being studied with increasing interest because of their ability to decrease emissions of unburned hydrocarbons, soot, and carbon dioxide;^1–3^ however, utilizing them may also promote the formation of some carbonyl compounds considered to be toxic intermediates, such as methyl vinyl ketone, acetaldehyde, and formaldehyde.^4^ Therefore, it is necessary to develop detailed models based on elementary reactions with well-defined reactant or product species to better utilize biofuels depending on the conditions. Despite the popularity of biofuels, reaction kinetics studies mainly focus on slightly oxygenated molecules (including 3 to 20 carbon atoms and very few oxygen atoms^5^) because of the limited reliable data available for the reaction kinetics of highly oxygenated molecules.

As one of the most common oxygenated molecules, ketones are extensively used in paints, industrial solvents,^6^ intermediate products or fuels in combustion processes, and “fuel tracers” for temperature measurement.^7^ Thus it is essential to explore and clarify the reactivity of ketones for developing detailed kinetic models of highly oxygenated molecules suitable for application in combustion regimes.^8^

Hydrogen atom abstraction reactions by small radicals such as H, CH_3_, OH and HO_2_ from fuel molecules always play an important role in the oxidation of fuels.^9^ It has been experimentally and theoretically proven that abstraction by OH radicals will result in the formation of water (H_2_O) and radicals.^10,11^ However, experimental evidence suggests that the rate constants of ketone + OH are larger than those of alkenes + OH. Meanwhile, β-position hydrogen atoms are relatively more likely to be abstracted than α-position ones.^12^ Consequently, a short-lived pre-reaction complex with a six-member^13^ or seven-member ring^14^ structure that can either decompose to reactants (ketone + OH) or form products (ketone radicals + H_2_O) has been proposed, and their contribution to the curvature of the Arrhenius plot has been discussed. Moreover, negative temperature dependence^15^ has been proposed to be the characteristic of the reaction with pre-reaction complexes, but Caralp *et al.*^16^ considered that to be the result of tunneling. Zhou *et al.*^17^ believed both the pre-reaction complex and tunneling could contribute to the difference in mechanism and kinetics between ketone + OH and the corresponding alkanes.

More investigations on the pre-reaction complex are needed as not all studies are in agreement. Considering that 2-butanone (MEK) is among the smallest ketones with an asymmetric structure, we carried out a theoretical study of the MEK + OH reaction by characterizing the potential energy surfaces (PESs) at the DLPNO-CCSD(T)/aug-cc-pVTZ//M06-2x-D3/may-cc-pVTZ level containing the corresponding pre- and post-reaction complexes. Wavefunction analyses were conducted to investigate the interactions between OH and MEK. Three mechanisms were compared based on rate constants, branching ratios, and the predicted reactant and product distribution to investigate the role of complexes, which proves to be a significant conclusion in this work.

## 2. Method

The lowest energy conformer of MEK with a trans CCCC dihedral angle was used following the result of Zhou *et al.*,^18^ since they proposed that the α- and β-hydrogen atom abstractions are independent of another chiral gauche conformer which contributes to the extent of 19.4%. *Ab initio* calculations were performed using in Gaussian 16 program.^19^ The equilibrium geometries and vibrational characteristics of stationary points (monomers, reactant and product complexes, transition states) were calculated at the dispersion corrected density functionals M062x-D3 (ref. 20–22) with convergent partially augmented basis sets may-cc-pVTZ^23–25^ to increase basis set superposition error (BSSE) for better description for weakly bonded complexes. Scaling factor of 0.9490 (ref. 26) was introduced for zero-point vibrational energy calculations at 298 K. The single point energies were corrected using the aug-cc-pVTZ basis sets and DLPNO-CCSD(T) method (default setting, TightPNO) in ORCA^27^ software, which were possible to approach the canonical CCSD(T) results within 1 kJ mol^−1^.^28,29^ The number of imaginary harmonic frequencies (0 or 1) has been checked for minimum or a transition state subjected to intrinsic reaction coordinate (IRC) calculations and the keyword downhill was used to find the corresponding pre- and post-complexes for further geometrical optimizations. Cartesian coordinates, frequencies and T1 diagnostics were provided in the supplemental material.

To study the interaction properties between OH and MEK of the pre-reaction complexes and transition states, wavefunction analyses were conducted using the Multiwfn^30^ program. The Non-Covalent Interactions (NCI) index^31^ based on the normalized and dimensionless reduced density gradient,^32^ was applied to obtain a chemically intuitive description to visualize the hydrogen bonding between OH and MEK in the complex structure in a three-dimensional space. Moreover, changes in the electronic structure before and after the reaction could be reflected through the density of states^33^ (DOS) distribution to demonstrate the effect of OH fragment on the HOMO energy level complex and transition state.

Rate constant calculations were carried out with the Mesmer^34^ program. The variational RRKM theory^35,36^ was employed for the complex-forming reactions and the transition state theory (TST)^37,38^ was used for the abstraction processes in the temperature of 250–2000 K with Eckart tunneling correlations.^39^ For the complex-forming reactions, the relaxed PES scan with geometry optimization at each point has been performed to search the loose transition state for VTST. Generalized internal coordinate of the distance between the geometric centers of OH and MEK has been scanned at 0.01 Å from the monomers to the formation of complexes. Low harmonic frequencies below 200 cm^−1^ that resemble torsions around single bonds were treated as hindered internal rotors. The energy transfer was modeled using <Δ*E*_down_> = 200 cm^−1^ (*T*/300)^0.85^ to represent the collision energy transfer probability *via* bath gas He, with the Lennard-Jones (LJ) parameters, *σ* = 2.55 Å and *ε* = 10.2 K, taken from literature values,^40^ and *σ* = 4.59 Å and *ε* = 450 K for C_4_H_9_O_2_ were based on the group additivity of the functional groups.^41^

## 3. Results and discussion

### 3.1 Potential energy surfaces and reaction mechanisms

**Fig. 1:**
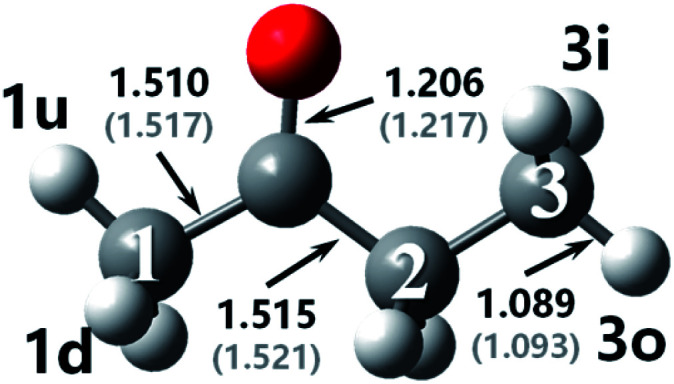
Optimized geometries for MEK at M06-2x-D3/may-cc-pVTZ; Zhou *et al.*^18^ at MP2/6-311G(d,p), round brackets.

**Fig. 2:**
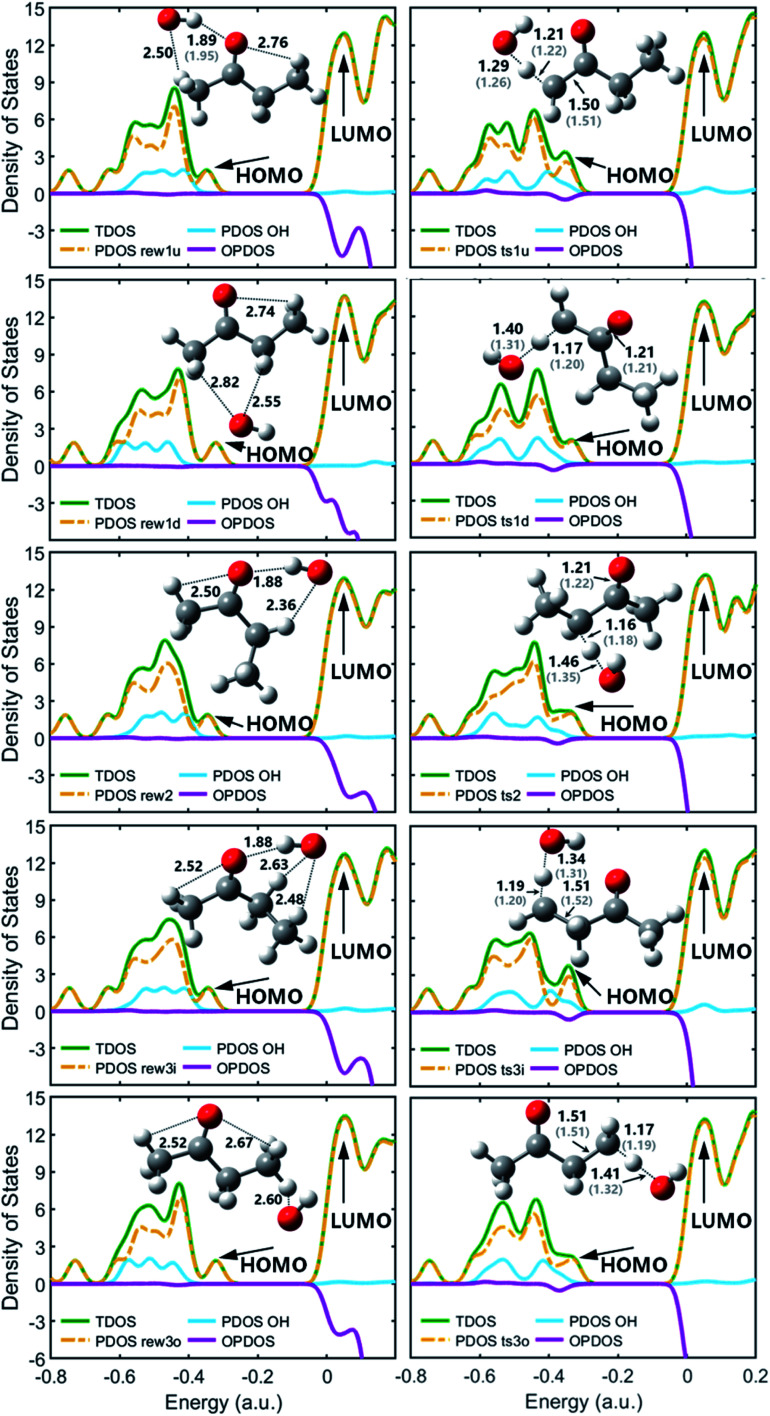
TDOS, PDOS and OPDOS maps with optimized geometries for the reactant complexes and transition states at M06-2x-D3/may-cc-pVTZ; Zhou *et al.*^18^ at MP2/6-311G(d,p), round brackets.

The optimized geometries at M06-2x-D3/may-cc-pVTZ compared with bond lengths in round brackets from Zhou *et al.*^18^ at MP2/6-311G(d,p) as well as the used nomenclature for the reactant and transition states in MEK + OH are displayed in Fig. 1 and 2. The figure shows that the difference in the calculation methods has little influence on the structure, for the configurations remain similar and bond lengths agree well with literature values,^18^ with the error being only ±1%. Numbers are used to distinguish carbon atoms in MEK, whereas numbers and letters are used to mark hydrogen atoms in different reaction sites as shown in Fig. 1. The letter w refers to the complex, which resembles wells in the PES. Therefore, rew1u represents the pre-reaction complex in the abstraction of the 1u hydrogen atom, whereas the prw1d represents the post-reaction complex of the corresponding abstraction channel.

As molecular structures do not easily depict intricate noncovalent interactions such as hydrogen bonding, which was determined to play an important role in the pre-reaction complex, visualization analyses based on wavefunction at M06-2x-D3/may-cc-pVTZ were conducted to better understand the interaction between OH and MEK. As is shown in Fig. 2, compared with covalent interactions, such as C–C bonds with a length of around 1.5 Å or C–H bonds with 1.2 Å, the distance action of the hydrogen bond interaction is in the range of 1.8–3 Å.

**Fig. 3:**
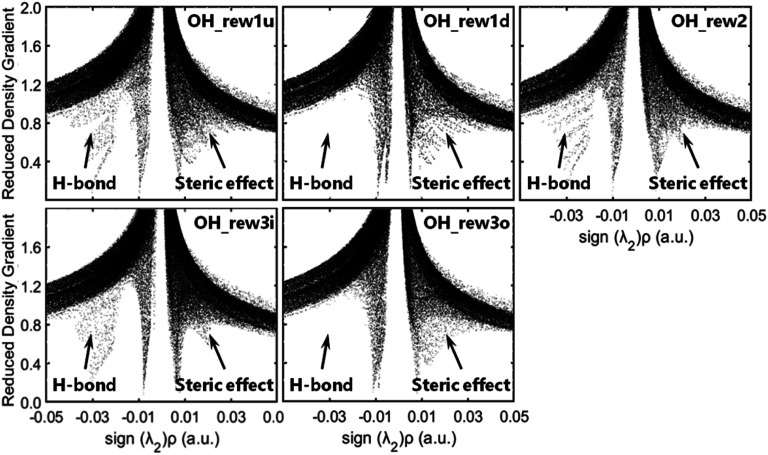
Reduced density gradient *versus* the electron density multiplied by the sign (*λ*_2_)*ρ*.

Δ_2_*ρ*, as the sign of the electron density, is often decomposed into three eigenvalues *λ*_i_ (Δ_2_*ρ* = *λ*_1_ + *λ*_2_ + *λ*_3_) along the three principal axes of maximal variation. The sign (*λ*_2_)*ρ* can be utilized to distinguish bonded (*λ*_2_ < 0) from non-bonded (*λ*_2_ > 0) interactions.^42^ The low-density, low-gradient spike remaining at negative values indicates the stabilizing effect of a hydrogen bond, whereas the low-density, low-gradient spike remaining at positive values indicates a sterically crowded structure, and the weak attraction with slightly negative values very near zero indicates van der Waals's force. As shown in Fig. 3, the pre-reaction complex structures such as rew1u, rew2 and rew3i were stabilized by stronger hydrogen bond forces, and the steric effect loading on rew3i was relatively weak. The structures of rew1d and rew3o have relatively weaker hydrogen bonds but higher steric effects, which may be more unstable. In other words, when OH radicals collide with the hydrogen atoms at the 1d or 3o site of MEK, the pre-reaction complexes may be more active.

Fig. 2 shows optimized geometries compared with Zhou *et al.*^18^ and the contributions of the OH and the MEK species to the frontier molecular orbitals (HOMO and LUMO) of the pre-reaction complexes and transition states by the total density of states (TDOS), partial density of states (PDOS), and overlap density of states (PDOS) maps. The OH species of pre-reaction complexes contribute almost nothing to the HOMO, but in transition states they play important roles in the HOMO, especially ts1u, ts2 and ts3i which show more stability with stronger hydrogen bond forces while OH segments in ts1d and ts3o contribute more to the LUMO. All OH species contribute most to the HOMO−1, both in pre-reaction complexes and transition states.

### 3.2 Reaction kinetics and fractional populations

Kinetics of abstraction channels were modeled by three different mechanisms, according to the role of pre- and post-reaction complexes. The general bimolecular reaction mechanism without complexes into consideration was modeled as A + B → TS → C + D shown in (I), while the mechanisms containing complexes were modeled shown in (II) and (III). In the mechanism (II), each abstraction channel was counted as a two-step mechanism. If *k*_1_ and *k*_−1_ are the forward and reverse rate constants for the first step and *k*_2_ corresponds to the second step, a steady-state analysis leads to a rate coefficient for each overall reaction channel which can be written as (1). In the mechanism (III), the process to form pre-reaction complexes seemed “displacement reactions” as the results of master equation for the pre-reaction complexes hard to sink as stable products. So, overall rate coefficients could be seen as one-step “well-skipping” reactions leading to the products.1
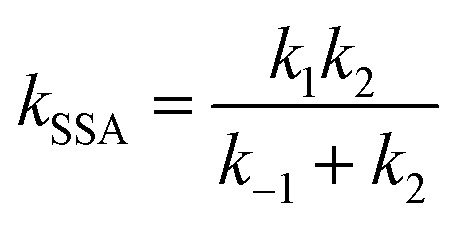


**Fig. 4:**
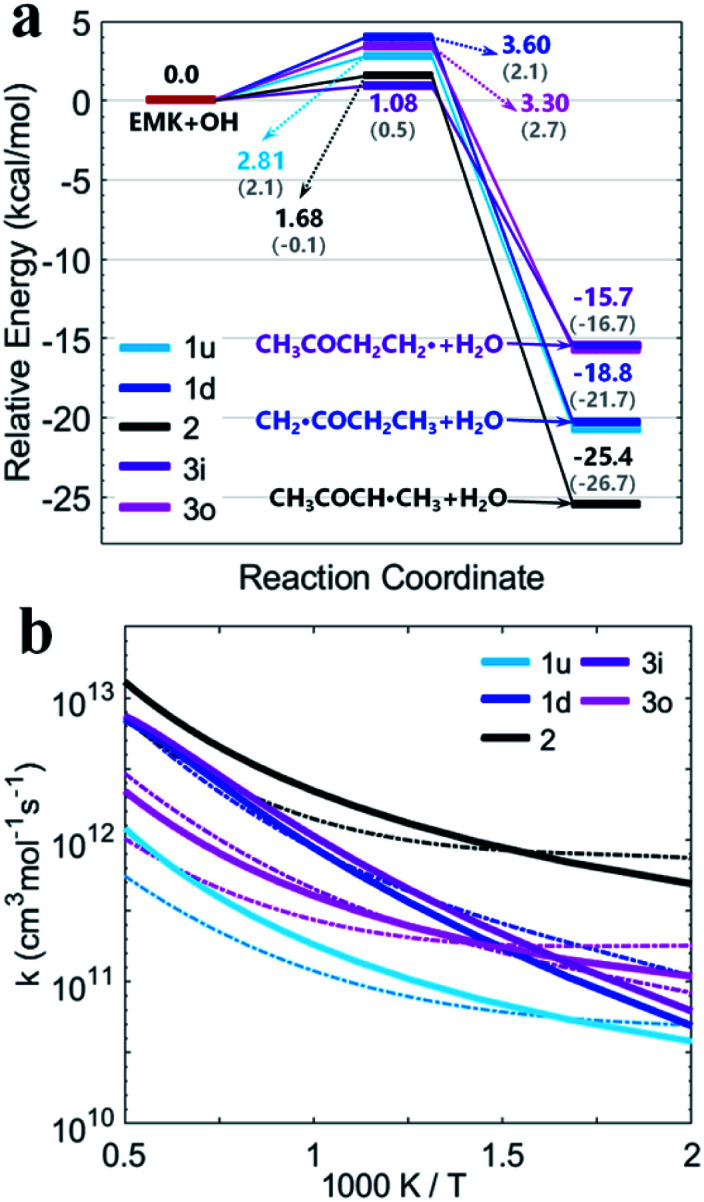
(a) PESs in kcal mol^−1^ for MEK + OH as the general bimolecular reaction mechanism at DLPNO-CCSD(T)/aug-cc-pVTZ//M06-2x-D3/may-cc-pVTZ; Zhou *et al.*^18^ at MP2/6-311G(d,p), round brackets. (b) Rate constants for the bimolecular reactions. Sign: this work, solid lines; Zhou *et al.*,^18^ chain dotted line.

Fig. 4(a) shows the PESs for hydrogen abstraction from MEK by OH considered as a general bimolecular reaction without pre- and post-reaction complexes at DLPNO-CCSD(T)/aug-cc-pVTZ//M06-2x-D3/may-cc-pVTZ. Three 2-butanoyl radicals formed *via* five transition states are CH_2_˙COCH_2_CH_3_, CH_3_COCH˙CH_3_ and CH_3_COCH_2_CH_2_˙. The products of abstraction at number 2 hydrogen site seem more stable than the hydrogen atoms of number 1 and 3 carbon atoms. For the transition states, as illustrated in Fig. 2, ts1u, ts2, and ts3i, whose species show more contributions to the HOMO, display more stable structures in lower zero-point energy compared with ts3o and ts1d. The results of rate constants obtained from the zero-point energy barriers between MEK + OH and corresponding transition states was shown in Fig. 4(b), compared with the result of Zhou *et al.*^18^ The relative error was in the range of 2–5 times in the same order of magnitude. The reaction channel 2 with a more stable transition state ts2 and the most stable product CH_3_COCH˙CH_3_ + H_2_O remains the most reactive in the range of 500–2000 K. However, the channels 1d and 3o, whose OH species contributed relatively less to the HOMO of the transition states ts1d and ts3o in Fig. 2, showed more reactivity, especially when the temperature was over 700 K.

**Fig. 5:**
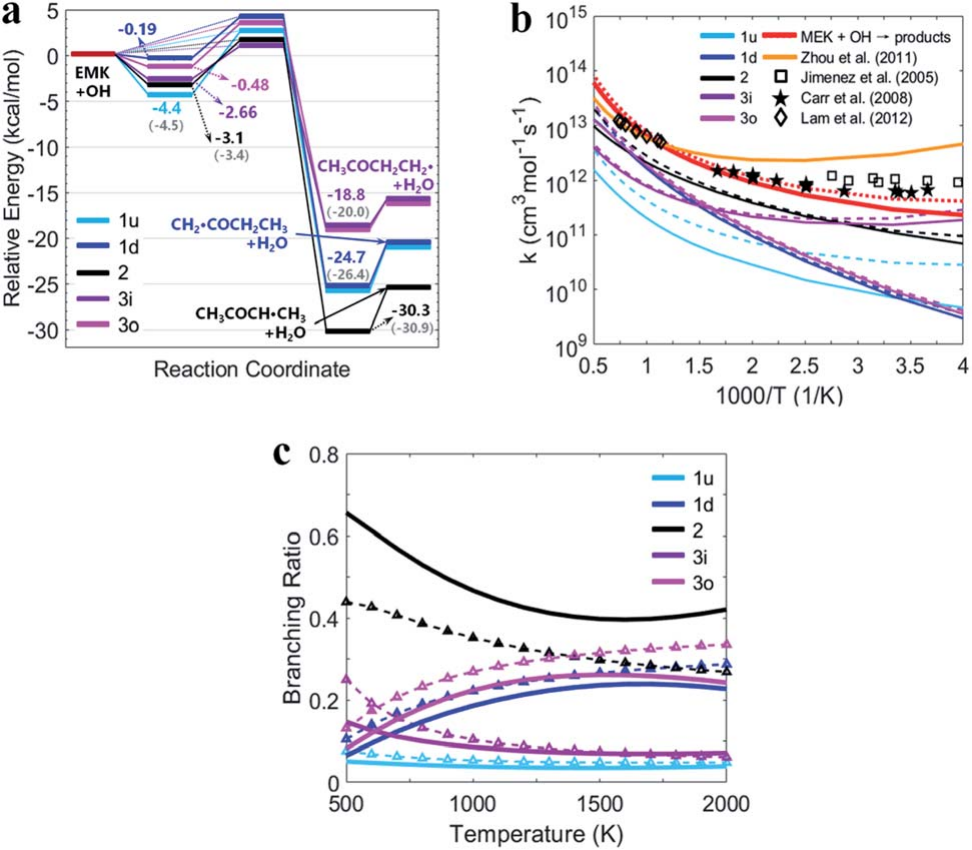
(a) PESs in kcal mol^−1^ for MEK + OH with complexes at DLPNO-CCSD(T)/aug-cc-pVTZ//M06-2x-D3/may-cc-pVTZ. Sign: the two-step mechanism, solid lines; the one-step “well-skipping” mechanism, dotted line; Zhou *et al.*^18^ at MP2/6-311G(d,p), round brackets. (b) Rate constants with complexes from this work compared with previous experimental data. Sign: total rate constants from this work, red bold solid line and dotted line. Total rate constants from Zhou *et al.*,^18^ orange bold solid line. (c) Predicted branching ratios *versus* temperature between 500–2000 K. Sign: the general bimolecular reaction mechanism, bold solid lines; the two-step mechanism, dotted lines marked with triangle. Colors refer to individual channels shown in PESs.

Fig. 5(a) shows the PESs for MEK + OH channels considering the pre- and post-reaction complexes at DLPNO-CCSD(T)/aug-cc-pVTZ//M06-2x-D3/may-cc-pVTZ level. Five pre-reaction complexes (rew1d, rew1u, rew2, rew3i and rew3o) in the entrance and four post-reaction complexes (prw1d, prw1u, prw2, prw3i and prw3o sharing the same structure) in the exit were found. The pre-reaction complexes are suspected to be formed when the OH radicals came close to MEK so that they both collided at the particular reactive site to form the corresponding transition states. However, with the presence of bath gas, such closure for collision may not be reactive, and the pre-reaction complex can separate back to OH and MEK. As previously discussed, the OH species in the complex structures contributes nothing to the HOMO but only tends to participate in the orbital mixture depending on the electron or states density analyses. The pre-reaction complexes with stronger hydrogen bonds such as rew1u, rew2 and rew3i apparently have more stable structures with lower zero-point energy than rew3o and rew1d. The stability of post-reaction complexes is of the same order with that of the product of three 2-butanoyl radicals.

**Scheme 1:**
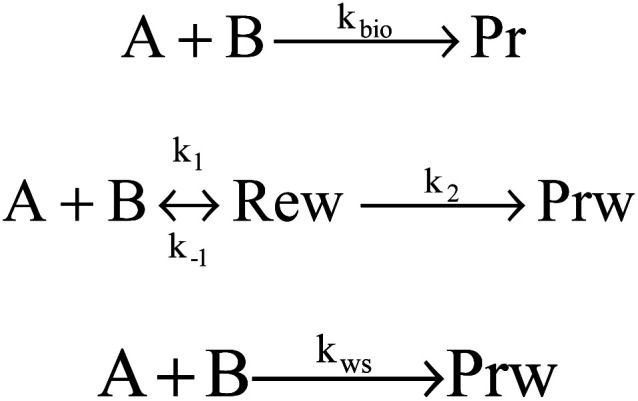
MEK + OH mechanisms considered in this work.

Fig. 5(b) presents the temperature dependence of the predicted individual high-pressure-limit rate constants of the PESs with complexes shown in Fig. 5(a). In general, results of rate constant calculations based on the mechanism (II) and (III) shown in Scheme 1 are in close accord with each other within 2 times, except that channel 1u with the most stable pre-reaction complex of the mechanism (II) about 4–10 times faster in 250–400 K than that of the mechanism (III).

For the overall rate constants of MEK + OH → products, the Arrhenius plot was shown in the range of 250–2000 K in Fig. 5(b). Results based on the mechanism (II) are faster than mechanism (III) ones by a factor about 1.5, while are faster than values from Zhou *et al.*^18^ by a factor about 2 at 1500 K and slower by a factor about 0.3 at 300 K. Kinetic measurements were performed by different researchers in different temperature range, as Lam *et al.*^43^ over 870–1360 K, Bader *et al.*^42^ over 970–1530 K, Tranter *et al.*^44^ over 300–440 K, Carr *et al.*^45^ over 220–600 K, Wallington *et al.*^46^ over 240–440 K, Calvé *et al.*^47^ over 240–370 K and so on. Their measurements are in agreement with each other in corresponding temperature ranges, and pressure dependence hasn't been stressed at their experimental conditions. The theoretical values based on the mechanism (II) and the mechanism (III) are consistent with that obtained in the experiments within about ±2 times over 250–2000 K, and predict less obvious negative temperature dependence than Zhou *et al.*^18^ High-pressure-limit total rate constants (cm^3^ mol^−1^ s^−1^) fitted as three-parameter Arrhenius expressions in the temperature range from 250 K to 2000 K are:

2 *k*_SSA_ = 3.477 × *T*^4^ × exp(834.3/*T*)

3 *k*_WS_ = 67.61 × *T*^3.6^ × exp(525.0/*T*)

Fig. 5(c) presents the predicted temperature dependence of branching ratios whether the complexes taken into account from 500 K to 2000 K. Channel 2 with relatively lower energy barrier and more stable products remains the most competitive one below 1300 K both in the general bimolecular reaction mechanism and the two-step mechanism. Channel 3o and 1d with more active pre-reaction complexes become more competitive when the temperature rises above 1300 K, but contribute less when the temperature is below 750 K. Channel 1u and 3i remain the two that contribute the least whether considering complexes or not, especially above 1300 K. In general, complexes make channels of MEK + OH contribute more equally. Because at higher temperature, competitiveness of channels with higher energy barriers and more active pre-reaction complexes may be enhanced, while at lower temperature, contributions of channels with lower energy barriers and more stable pre-reaction complexes with stronger hydrogen bonds could be improved.

**Fig. 6:**
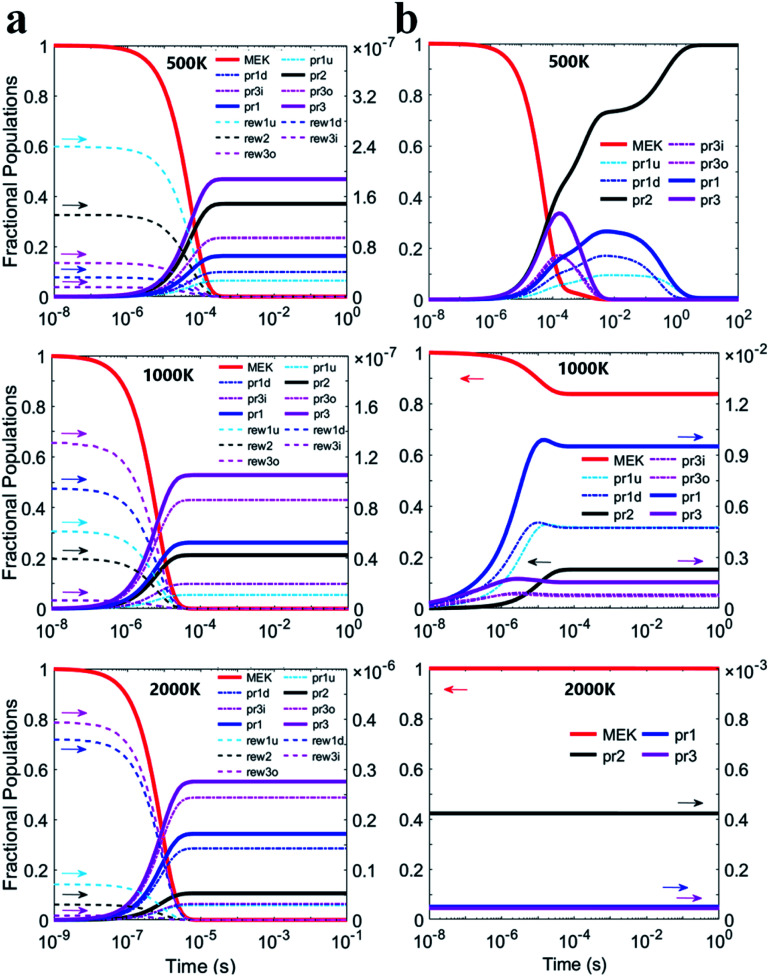
Fraction populations of the reactants and the products *versus* time at 500 K, 1000 K and 2000 K based on (a) the two-step mechanism and (b) the general bimolecular reaction mechanism.

Fig. 6(a) and (b) illustrate predicted fraction populations of the reactants and the products *versus* time whether the complexes taken into account. The population of MEK was considered as 1 before the reaction. Three 2-butanoyl radicals (CH_2_˙COCH_2_CH_3_, CH_3_COCH˙CH_3_ and CH_3_COCH_2_CH_2_˙) were named pr1, pr2 and pr3 marked with bold lines. For the two-step mechanism as shown in Fig. 6(a), the reactants of all channels would be completely consumed in about 0.1 milliseconds at 500 K, 10^−2^ milliseconds at 1000 K and 10^−3^ milliseconds at 2000 K. Populations of pre-reaction complexes exhibit the same trend as that of the reactant monomers differing by 7 orders of magnitude. CH_3_COCH_2_CH_2_˙ radicals (pr3) remain the most products and are expected to be half of the total consumption of MEK. For the he general bimolecular reaction mechanism shown in Fig. 6(b), the reactants of all channels would be almost completely consumed in about 0.1 milliseconds at 500 K, whereas at 1000 K, less than 20% of the reactants may be consumed and at 2000 K, the reactants could scarcely be consumed for the competition of reversible reaction channels.

## 4. Conclusions

Hydrogen abstractions from MEK molecules by OH radicals were investigated based on the detailed PESs at DLPNO-CCSD(T)/aug-cc-pVTZ//M06-2x-D3/may-cc-pVTZ level. Five pre-reaction complex structures of the corresponding transition states were found at the entrance of the channels and four post-reaction complex structures were found at the exit. Theoretical calculations based on three kinds of mechanisms—(I) the general bimolecular reaction mechanism, (II) the two-step mechanism by the steady-state analysis and (III) the one-step “well-skipping” reaction mechanism, were discussed about the effects of complexes.

Results of the mechanism (II) and the mechanism (III) are in well consistent with each other within 2 times. Overall, the consideration of complexes shows better agreement with experiments especially below 500 K and makes channels of MEK + OH contribute more equally compared with the mechanism (I). Predicted fraction populations of pre-reaction complexes exhibit the same trend but 7 magnitudes less than that of the reactant monomers. The reactants are expected to be consumed completely when the complexes taken into account, while scarcely react at high temperature in the mechanism (I).

## Author contributions

Yi Gao: methodology, investigation, writing – original draft. Yang Zhao: investigation, writing – original draft. Qingbao Guan: supervision, resources – original draft, writing – review & editing. Fuke Wang: supervision – original draft. All authors analyzed and interpreted the data.

## Conflicts of interest

The authors declare no conflict of interest.

## Supplementary Material

RA-010-D0RA05332E-s001
