# Supplementary material for: Ab initio kinetics predictions for the role of pre-reaction complexes in hydrogen abstraction from 2-butanone by OH radicals
Source: RSC Adv. 2020 Sep 8;10(55):33205–12. doi: 10.1039/d0ra05332e (PMC9088179; doi:10.1039/d0ra05332e)
Supplement: RA-010-D0RA05332E-s001 [file RA-010-D0RA05332E-s001.pdf]

Electronic Supplementary Material (ESI) for RSC Advances.
This journal is © The Royal Society of Chemistry 2020

Appendix for:

Ab initio kinetics predictions for the role of pre-reaction complexes in hydrogen abstraction from 2-butanone by OH radical

Yi Gao,[a] Yang Zhao,[b] Qingbao Guan,[c] and Fuke Wang.[b]*

[a]Center for Combustion Energy and Key Laboratory for Thermal Science and Power Engineering of MOE, Tsinghua University, Beijing, 100084, China

[b]Soft Materials, Institute of Materials Research and Engineering, Agency for Science, Technology and Research (A*STAR), 2 Fusionopolis Way, #08-03 Innovis, Singapore 138634

[c]State Key Laboratory for Modification of Chemical Fibers and Polymer Materials, International Joint Laboratory for Advanced Fiber and Low-dimension Materials, College of Materials Science and Engineering, Donghua University, Shanghai, 201620, P. R. China.

To be submitted to *RSC Advances*

* Corresponding author E-mail: y-g18@mails.tsinghua.edu.cn (Yi Gao)

Table of Contents:

Table 1. Cartesian coordinates (in Å) and frequencies of the optimized geometries at M06-2x-D3/may-cc-pVTZ, T1 diagnostics at DLPNO-CCSD(T)/aug-cc-pVTZ

MEK S3
rew1u S3
rew1d S4
rew2 ....S4
rew3i S5
rew3o S5
ts1u S6
ts1d S6
ts2 ....S7
ts3i S7
ts3o S8
prw1u S8
prw1d S9
prw2 ....S9
prw3i S10
pr01 ....S10
pr02 ....S11
pr03 ....S11

Table 1. Cartesian coordinates (in Å) and frequencies of the optimized geometries at M06-2x-D3/may-cc-pVTZ, T1 diagnostics at DLPNO-CCSD(T)/aug-cc-pVTZ.

MEK

T1 diagnostics: 0.012174707	Harmonic frequencies:	
Cartesian coordinates:	24.84 1012.85 1496.51	56.22 1012.85 1496.51
C 0.52269100 0.16841900 0.00833900	107.97 1116.57 1503.95	126.80 1116.57 1503.95
O 0.40152300 1.36787100 0.00924900	212.88 1137.86 1845.34	219.42 1137.86 1845.34
C 1.87945900 -0.49377300 -0.01369800	253.52 1201.16 3052.36	253.52 1201.16 3052.36
H 1.99647500 -1.05219400 -0.94398100	406.35 1285.53 3067.97	406.35 1285.53 3067.97
H 2.66050200 0.25669200 0.06263800	475.41 1375.77 3079.78	475.41 1375.77 3079.78
H 1.96500600 -1.21070600 0.80358500	597.35 1399.98 3082.07	597.35 1399.98 3082.07
C -0.67622500 -0.75709900 0.02384000	755.35 1418.71 3131.88	755.35 1418.71 3131.88
H -0.57092300 -1.44885900 -0.81689800	781.15 1456.34 3152.82	781.15 1456.34 3152.82
H -0.59366200 -1.37907400 0.92017600	949.99 1470.57 3154.92	949.99 1470.57 3154.92
C -2.00296500 -0.02127500 -0.01962800	955.61 1481.21 3180.93	955.61 1481.21 3180.93
H -2.83511000 -0.72293000 -0.00221100		*(With hindered internal*
H -2.09560400 0.65184800 0.83044300		*rotation analysis)*
H -2.07663400 0.58462000 -0.92086100		

rew1u

T1 diagnostics: 0.013170465	Harmonic frequencies:	Harmonic frequencies:
Cartesian coordinates:	28.18 787.29 1491.8	34.22 787.29 1491.80
C -0.12834900 0.23209100 -0.06338500	83.97 949.82 1498.28	83.97 949.82 1498.28
O 0.27016400 -0.90932600 -0.14774100	104.97 994.85 1504.41	103.37 994.85 1504.41
C 0.83278600 1.39398400 -0.02005000	140.81 1014.77 1817.87	154.46 1014.77 1817.87
H 0.33563700 2.34546900 -0.19350400	161.24 1114.38 3049.1	161.24 1114.38 3049.10
H 1.29648400 1.41442400 0.96849000	220.38 1133.43 3067.5	225.56 1133.43 3067.50
H 1.62893400 1.24481100 -0.74833800	278.48 1199.73 3077.51	278.48 1199.74 3077.51
C -1.59928500 0.54695700 0.02516100	419.85 1285.35 3081.46	419.85 1285.35 3081.46
H -1.73909100 1.18554000 0.90301300	478.16 1381.93 3136.52	478.16 1381.93 3136.52
H -1.83660000 1.18701400 -0.83065000	485.73 1397.8 3154.7	485.73 1397.80 3154.70
C -2.48533200 -0.68340900 0.06995000	542.7 1421.03 3156.98	542.70 1421.03 3156.98
H -3.53442400 -0.40011800 0.12954500	601.67 1452.06 3169.21	601.67 1452.06 3169.21
H -2.33879400 -1.29418600 -0.81868600	752.18 1474.12 3571.75	752.18 1474.12 3571.75
H -2.24195500 -1.30115200 0.93244100		*(With hindered internal*
O 3.04974900 -0.62943100 0.09828900		*rotation analysis)*
H 2.15158700 -1.00948300 -0.01675500		

rew1d

T1 diagnostics: 0.012934953				Harmonic frequencies:					
Cartesian coordinates:				44.87	780.32	1479.39	44.87	780.32	1479.39
C	0.61732500	-0.68035900	-0.00000500	71.55	953.96	1493.8	58.61	953.96	1493.80
O	1.78721400	-0.97265800	-0.00008900	72.12	954.04	1502.19	72.12	954.04	1502.19
C	-0.47123000	-1.72276400	0.00006400	95.56	1011.37	1843.63	95.56	1011.37	1843.63
H	-1.11139800	-1.59102200	-0.87333000	139.34	1116.82	3039.22	159.59	1116.82	3039.22
H	-0.03278200	-2.71614700	-0.00001600	178.31	1139.83	3065.48	178.31	1139.83	3065.48
H	-1.11121100	-1.59108600	0.87360800	202.13	1201.5	3070.19	193.63	1201.50	3070.19
C	0.16224000	0.76667300	0.00004300	235.75	1281.88	3078.95	235.75	1281.88	3078.95
H	-0.48479300	0.89882800	-0.87346200	257.82	1371.44	3133.06	257.82	1371.44	3133.06
H	-0.48473200	0.89878800	0.87359600	409.58	1401.86	3150.62	409.58	1401.86	3150.62
C	1.31087400	1.75911300	0.00002100	484.3	1417.56	3155.18	484.30	1417.56	3155.18
H	0.94092300	2.78325800	0.00005800	597.43	1459.04	3181.81	597.43	1459.04	3181.81
H	1.94013300	1.61584400	0.87634000	761.3	1469.51	3767.33	761.30	1469.51	3767.33
H	1.94006800	1.61588300	-0.87635200				*(With hindered internal*		
O	-2.83657000	0.46240000	-0.00006000				*rotation analysis)*		
H	-2.91662400	1.43173900	0.00000900						

rew2

T1 diagnostics: 0.013561083				Harmonic frequencies:					
Cartesian coordinates:				23.7	813.62	1494.85	23.70	813.62	1494.85
C	-0.33645600	-0.61857100	0.07245900	55.33	956.1	1509.54	55.33	956.10	1509.54
O	0.64392200	-1.28189100	-0.18638400	75.92	960.94	1511.12	75.92	960.94	1511.12
C	-1.71182000	-1.22970400	0.02446900	156.66	1024.52	1821.08	156.66	1024.52	1821.08
H	-2.22328600	-0.87938400	-0.87424400	178.76	1103.86	3055.7	178.76	1103.86	3055.70
H	-1.63208700	-2.31186200	-0.00930000	224.36	1133.91	3068.81	249.84	1133.91	3068.81
H	-2.30935800	-0.91233400	0.87920800	278.9	1247.04	3073.07	278.90	1247.04	3073.07
C	-0.23321600	0.83882800	0.45663300	417.2	1303.06	3119.19	417.20	1303.06	3119.19
H	-0.43266000	0.89309100	1.53136500	486.94	1358.31	3133.17	486.94	1358.31	3133.17
H	0.79410900	1.16558800	0.29915300	537.43	1395.07	3145.22	537.43	1395.07	3145.22
C	-1.21753900	1.73656100	-0.29193400	563.74	1417.6	3152.88	563.74	1417.60	3152.88
H	-1.06956700	2.77524900	-0.00406000	753.84	1464.54	3185.74	753.84	1464.54	3185.74
H	-1.07007700	1.66399100	-1.36930500	772.83	1475.2	3579.45	772.83	1475.20	3579.45
H	-2.25114500	1.47349200	-0.07064700				*(With hindered internal*		
O	2.96425200	0.30656200	-0.03849900				*rotation analysis)*		
H	2.32286600	-0.42788300	-0.15286600						

rew3i

T1 diagnostics: 0.013150719				Harmonic frequencies:					
Cartesian coordinates:				46.56	921.39	1500.43	46.56	921.39	1500.43
C	-0.76366900	-0.37947500	0.10850000	91.99	983.38	1506	91.99	983.38	1506.00
O	-0.11900700	-1.35897700	-0.19978900	145.22	994.41	1598.66	145.22	994.41	1598.66
C	-2.24927800	-0.32120100	-0.10962500	165.76	1081.99	1834	165.76	1081.99	1834.00
H	-2.51187500	0.59873800	-0.63448200	174.12	1110.59	3064.71	177.70	1110.59	3064.71
H	-2.58852100	-1.18889000	-0.66676200	221.46	1157.39	3068.34	221.46	1157.39	3068.34
H	-2.74680200	-0.28485300	0.86155000	249.46	1235.95	3092.11	249.46	1235.95	3092.11
C	-0.09793300	0.84734100	0.68505100	397.59	1291.84	3126.99	397.59	1291.84	3126.99
H	-0.82375200	1.43858600	1.24326100	512.47	1365.73	3142	512.47	1365.73	3142.00
H	0.69473400	0.52581200	1.36001100	542.31	1396.04	3150.02	542.31	1396.04	3150.02
C	0.50337600	1.68119400	-0.45331700	606.16	1420.52	3186.21	606.16	1420.52	3186.21
H	0.98201600	2.57286900	-0.05423900	781.41	1463.92	3218.72	781.41	1463.92	3218.72
H	1.25464700	1.10457000	-0.99221000	803.37	1477.65	3561.53	803.37	1477.65	3561.53
H	-0.26352200	1.99599700	-1.16187000				*(With hindered internal*		
O	2.60732600	-0.71120600	0.03832900				*rotation analysis)*		
H	1.74154800	-1.16852100	-0.04723000						

rew3o

T1 diagnostics: 0.012838113				Harmonic frequencies:					
Cartesian coordinates:				27.12	783.53	1480.65	44.72	783.53	1480.65
C	1.17736000	0.05908400	-0.10183500	34.88	950.17	1502.28	67.73	950.17	1502.28
O	1.37408200	0.99259500	-0.83997900	76.69	957.77	1505.64	76.69	957.77	1505.64
C	2.19802200	-1.03749600	0.08275600	81.06	1014.77	1842.19	81.06	1014.77	1842.19
H	1.73847800	-2.01357300	-0.07569700	117.13	1117.17	3057.61	125.33	1117.17	3057.61
H	3.02594500	-0.89507700	-0.60525000	129.35	1136.15	3069.17	129.35	1136.15	3069.17
H	2.56752500	-1.01931200	1.10949300	208.27	1204.01	3069.62	208.27	1204.01	3069.62
C	-0.10476700	-0.07789100	0.69034400	260.04	1287.32	3088.89	260.04	1287.32	3088.89
H	-0.58657800	-1.00705700	0.37284800	279.13	1376.64	3133.04	279.13	1376.64	3133.04
H	0.16321100	-0.23171800	1.73931900	409.04	1400.28	3137.82	409.04	1400.28	3137.83
C	-1.03966200	1.10574400	0.52028500	475.02	1412.96	3144.99	475.02	1412.96	3144.99
H	-1.96529000	0.94799400	1.07309800	598.56	1454.99	3181.72	598.56	1454.99	3181.72
H	-0.57503100	2.02412600	0.87567900	753.76	1471.84	3772.64	753.76	1471.84	3772.64
H	-1.26933500	1.25675900	-0.53424600				*(With hindered internal*		
O	-3.02174000	-0.89435600	-0.42595800				*rotation analysis)*		
H	-3.30337600	-0.14471100	-0.97704600						

ts1u

T1 diagnostics: 0.019259890				Harmonic frequencies:					
Cartesian coordinates:				-1466.92	815.45	1454.9	-1466.92	815.45	1454.90
C	0.09734400	0.10361100	0.09092100	33.07	906.17	1480.51	41.06	906.17	1480.51
O	-0.11994200	-1.07695000	0.22733000	86.71	951.33	1496.75	92.45	951.33	1496.75
C	-1.00735400	1.12137600	0.16077700	147.13	1007.26	1504.11	147.13	1007.26	1504.11
H	-0.90403000	1.94089000	-0.54676800	217.11	1015.29	1820.52	223.84	1015.29	1820.52
H	-2.04435300	0.54273800	-0.09297200	254.45	1119.63	3053.25	254.45	1119.63	3053.25
H	-1.13659300	1.49467100	1.17603900	280.84	1125.84	3081.12	280.84	1125.84	3081.12
C	1.49069500	0.64086700	-0.14266700	365.38	1220.83	3082.86	365.38	1220.83	3082.86
H	1.47721900	1.13487900	-1.11915000	435.74	1223.44	3103.45	432.35	1223.44	3103.45
H	1.66307700	1.44196500	0.58185100	482.23	1287.74	3154.6	482.23	1287.74	3154.60
C	2.56584100	-0.42825500	-0.07320400	585.28	1381.28	3157.98	585.28	1381.28	3157.98
H	3.54808300	0.00350300	-0.25573800	704.36	1418.35	3177.01	704.36	1418.35	3177.01
H	2.57128000	-0.90538700	0.90506100	751.5	1432.52	3730.56	751.50	1432.52	3730.56
H	2.38184000	-1.20468100	-0.81288400				*(With hindered internal*		
O	-2.90036500	-0.41515300	-0.22828100				*rotation analysis)*		
H	-2.27322100	-1.13734900	-0.04278800						

ts1d

T1 diagnostics: 0.019310363				Harmonic frequencies:					
Cartesian coordinates:				-867.67	795.75	1455.3	-867.67	795.75	1455.30
C	-0.25382100	0.63562500	0.07344200	37.69	953.2	1460.96	47.09	953.20	1460.96
O	-0.85871100	1.46836700	-0.55451000	57.14	976.59	1496.12	57.14	976.59	1496.12
C	1.15531600	0.89230200	0.53588600	97.02	1017.64	1504.2	76.27	1017.64	1504.20
H	1.80286800	0.09170200	-0.02661900	108.98	1052.57	1832.81	132.53	1052.57	1832.81
H	1.51134300	1.87613800	0.24718900	208	1117.33	3053.49	213.13	1117.33	3053.49
H	1.29435500	0.68657800	1.59571500	250.61	1154.16	3080.46	250.61	1154.16	3080.46
C	-0.83867400	-0.71126400	0.42610300	293.94	1209.23	3084.05	293.94	1209.23	3084.05
H	-0.14214200	-1.46903900	0.05529300	406.68	1282.43	3111.87	406.68	1282.43	3111.87
H	-0.81257000	-0.80383900	1.51606200	485.15	1311.32	3154.17	485.15	1311.32	3154.17
C	-2.24119000	-0.91434600	-0.11698000	584	1385.64	3154.64	584.00	1385.64	3154.64
H	-2.62193400	-1.89597900	0.15890500	694.78	1393.9	3199.64	694.78	1393.90	3199.64
H	-2.91856400	-0.15562300	0.27047100	779.65	1419.21	3793.3	779.65	1419.21	3793.30
H	-2.24721500	-0.83130300	-1.20220700				*(With hindered internal*		
O	2.58742100	-0.98017900	-0.47043100				*rotation analysis)*		
H	3.37439800	-0.81804000	0.07401100						

ts2

T1 diagnostics: 0.018869895				Harmonic frequencies:					
Cartesian coordinates:				-805.94	831.6	1479.76	-805.94	831.60	1479.76
C	-0.81783100	-0.27404700	0.05967700	34.12	934.58	1485.37	53.20	934.58	1485.37
O	-1.01057700	-0.95807600	1.03517900	69.79	958.29	1493.76	69.79	958.29	1493.76
C	-1.85996700	0.66181300	-0.49292700	112.56	1018	1562.23	112.56	1018.00	1562.23
H	-2.16525700	0.32113000	-1.48378000	129.84	1087.83	1833.44	144.90	1087.83	1833.45
H	-2.72254800	0.69104300	0.16578300	185.76	1118.1	3066.55	188.87	1118.10	3066.55
H	-1.43302000	1.65906900	-0.60800700	194.86	1179.63	3072.1	197.25	1179.63	3072.10
C	0.51339200	-0.28566300	-0.65140500	255.97	1219.2	3098.09	255.97	1219.20	3098.09
H	0.38058200	-0.20363800	-1.73186700	404.16	1328.52	3132.48	404.16	1328.52	3132.48
H	0.96785300	0.74755100	-0.38219800	456.71	1379.75	3138.65	456.71	1379.75	3138.65
C	1.45619500	-1.38611500	-0.23053100	597.08	1403.01	3160.27	597.08	1403.01	3160.27
H	2.42948400	-1.26596000	-0.70182800	672.36	1417.46	3182.49	672.36	1417.46	3182.49
H	1.58084900	-1.39299900	0.85060400	782.24	1468.84	3775.19	782.24	1468.84	3775.19
H	1.05187800	-2.35888100	-0.51313700				*(With hindered internal*		
O	1.35803500	1.95205400	0.34749100				*rotation analysis)*		
H	1.37978200	1.55493200	1.23418100						

ts3i

T1 diagnostics: 0.019350922				Harmonic frequencies:					
Cartesian coordinates:				-1110.13	853.79	1453.32	-1110.13	853.79	1453.32
C	-0.90236800	-0.15053500	-0.10213000	44.72	934.39	1463.49	44.72	934.39	1463.49
O	-0.38934200	-1.09152000	-0.65971300	95.75	974.19	1467.92	118.55	974.19	1467.92
C	-2.35336500	-0.16610100	0.30153600	122.3	983.84	1480.41	122.30	983.84	1480.41
H	-2.90745600	0.51976300	-0.34219000	154.28	1036.89	1834.09	154.28	1036.89	1834.09
H	-2.75929500	-1.16751200	0.19615100	246.88	1126.94	3044.16	246.88	1126.94	3044.16
H	-2.47030500	0.18790200	1.32586300	344.68	1140.43	3068.71	344.68	1140.43	3068.71
C	-0.12836100	1.10999500	0.22435400	402.26	1204.81	3078.14	402.26	1204.81	3078.14
H	-0.73505500	1.96495700	-0.09283600	442.37	1257.1	3111.3	442.37	1257.10	3111.30
H	-0.07741200	1.17833000	1.31528000	471.41	1317.71	3133.74	471.41	1317.71	3133.74
C	1.24918000	1.15756900	-0.38393400	596.24	1379.03	3183.83	596.24	1379.03	3183.83
H	1.80805500	2.04981600	-0.11261700	715.58	1404.35	3185.56	715.58	1404.35	3185.56
H	1.89216900	0.26022400	0.06371800	762.04	1436.97	3744.08	762.04	1436.97	3744.08
H	1.25173600	0.99800300	-1.45921400				*(With hindered internal*		
O	2.29019500	-0.94097200	0.51322800				*rotation analysis)*		
H	1.60021800	-1.43711000	0.03877000						

ts3o

T1 diagnostics: 0.019076714				Harmonic frequencies:					
Cartesian coordinates:				-744.05	830.38	1457.45	-744.05	830.38	1457.45
C	1.32726900	0.10153900	-0.02532200	40.25	938.39	1470.47	56.13	938.39	1470.47
O	1.57212100	1.27984300	-0.08511600	54.08	952.11	1480.52	54.08	952.11	1480.52
C	2.41517200	-0.94362600	-0.01062600	63.79	1020.13	1482.82	77.50	1020.13	1482.82
H	2.21543900	-1.71385200	-0.75596800	85.2	1085.61	1842.1	85.20	1085.61	1842.10
H	3.37871700	-0.47861600	-0.19617000	115.07	1134.76	3060.49	133.89	1134.76	3060.49
H	2.42785700	-1.43468800	0.96398300	220.7	1196.62	3069.64	220.70	1196.62	3069.64
C	-0.09772300	-0.41884900	0.04460100	320.74	1252.2	3094.64	320.74	1252.20	3094.64
H	-0.26703100	-1.02923000	-0.84692600	397.31	1281.99	3113.36	397.31	1281.99	3113.36
H	-0.16682200	-1.10917400	0.88893300	473.42	1317.08	3133.64	473.42	1317.08	3133.64
C	-1.12197100	0.68303600	0.14669900	594.08	1369.92	3180.18	594.08	1369.92	3180.18
H	-2.16970700	0.16762700	0.15997900	705.11	1394.21	3183	705.11	1394.22	3183.00
H	-1.05565600	1.24901300	1.07287100	755.06	1419.81	3796.87	755.06	1419.81	3796.87
H	-1.10096700	1.35815900	-0.70479900				*(With hindered internal*		
O	-3.39290100	-0.49840700	-0.03687600				*rotation analysis)*		
H	-3.83208200	0.20667800	-0.53808200						

prw1u

T1 diagnostics: 0.013922019				Harmonic frequencies:					
Cartesian coordinates:				42.99	752.26	1495.34	43.17	752.26	1495.34
C	0.21192400	0.32180300	-0.00006700	66.81	801.12	1504.29	71.06	801.12	1504.29
O	-0.28816900	-0.80183200	-0.00021400	111.99	819.77	1634.6	111.99	819.77	1634.60
C	-0.61300000	1.49989800	-0.00006400	144.58	994.72	1669.35	144.62	994.72	1669.35
H	-0.16374200	2.48215200	0.00015200	155.74	1031.58	3053.55	155.74	1031.58	3053.55
H	-3.67525300	-1.18651800	0.00061300	221.4	1083.95	3079.79	227.46	1083.95	3079.79
H	-1.68870200	1.39559500	-0.00028000	282.59	1113.61	3083.67	282.59	1113.61	3083.67
C	1.71453600	0.50802600	0.00011700	392.11	1239.97	3153.61	396.73	1239.97	3153.61
H	1.97226900	1.11791600	-0.87032400	412.05	1278.6	3154.33	412.05	1278.60	3154.33
H	1.97207700	1.11771100	0.87075800	428.31	1397.15	3166.91	428.31	1397.15	3166.91
C	2.47767300	-0.80374400	0.00004600	510.05	1418.01	3285.37	510.05	1418.01	3285.37
H	3.55110500	-0.62347000	0.00018200	582.98	1461.55	3696.65	583.74	1461.55	3696.65
H	2.22323000	-1.39668700	0.87658300	605.54	1486.6	3949.17	605.54	1486.60	3949.17
H	2.22342200	-1.39648100	-0.87668700				*(With hindered internal*		
O	-3.08415600	-0.43251900	0.00006900				*rotation analysis)*		
H	-2.18260700	-0.79130200	-0.00002400						

prw1d

T1 diagnostics: 0.019350922				Harmonic frequencies:					
Cartesian coordinates:				34.6	792.88	1503.23	34.60	792.88	1503.23
C	0.83280300	-0.44429000	0.12031400	63.8	807.57	1509.34	63.80	807.57	1509.34
O	0.09045600	-1.37357400	-0.20093100	104.21	817.06	1633.35	104.21	817.06	1633.35
C	2.24918500	-0.56225500	-0.08848900	133	990.2	1649.61	133.00	990.20	1649.61
H	-1.74319300	-0.93277900	-0.06548000	166.1	1004.48	3063.6	166.10	1004.48	3063.60
H	2.63553800	-1.47100600	-0.52440500	221.51	1075.57	3083.31	221.51	1075.57	3083.31
H	2.92607800	0.23603800	0.17608800	239.41	1114.01	3130.37	239.41	1114.01	3130.37
C	0.28063000	0.84543700	0.67662400	376.73	1249.73	3146.9	376.73	1249.73	3146.90
H	-0.59898800	0.61267700	1.27476500	385.01	1310.5	3157.78	383.83	1310.50	3157.78
H	1.02530800	1.32502400	1.31253100	420.05	1355.98	3178.76	420.05	1355.98	3178.76
C	-0.11569900	1.77463000	-0.47591700	538.18	1407.69	3295.01	538.18	1407.69	3295.01
H	-0.50491600	2.71362000	-0.08726200	581.65	1476.75	3694.98	581.65	1476.75	3694.98
H	0.74009400	1.99769600	-1.11482700	641.02	1489.59	3945.52	641.02	1489.59	3945.52
H	-0.89362300	1.30689300	-1.07688600				*(With hindered internal rotation analysis)*		
O	-2.56402500	-0.42907000	0.05240500						
H	-3.27926200	-1.04814000	-0.10150700						

prw2

T1 diagnostics: 0.014235692				Harmonic frequencies:					
Cartesian coordinates:				13.14	642.94	1480.17	38.57	642.94	1480.17
C	-0.96828600	-0.23413600	-0.00433300	44.57	833.51	1488.6	78.55	833.51	1488.60
O	-0.06396600	-1.06804600	-0.02453300	111.27	964.25	1642.34	111.27	964.25	1642.34
C	-2.41525600	-0.66150100	0.01095600	138.41	972.28	1674.73	138.41	972.28	1674.73
H	-2.94068300	-0.24735200	-0.85035800	151.23	1037.67	3046.3	160.30	1037.67	3046.30
H	-2.47550300	-1.74526500	-0.00975900	160.3	1083.98	3069.01	161.60	1083.98	3069.01
H	-2.90973900	-0.28373500	0.90676300	164.06	1128.8	3086.89	175.42	1128.80	3086.89
C	-0.67782600	1.17547200	0.00633500	255.24	1232.3	3131.85	255.24	1232.30	3131.85
H	-1.51125300	1.86527900	0.02747400	363.07	1383.52	3166.48	363.07	1383.52	3166.48
H	3.20716900	-1.55793800	0.01943900	419.55	1395.49	3183.7	419.55	1395.49	3183.70
C	0.71363500	1.67493400	-0.00796600	513.5	1463.8	3201.3	513.50	1463.80	3201.30
H	1.26924700	1.30040400	0.85661300	577.38	1466.77	3715.77	577.38	1466.77	3715.77
H	1.25678300	1.28605200	-0.87364900	610.84	1475.46	3945.37	610.84	1475.46	3945.37
H	0.75485600	2.76024700	-0.01603800				*(With hindered internal rotation analysis)*		
O	2.76624100	-0.70703000	0.01373500						
H	1.81732100	-0.90569400	-0.00405300						

prw3i & prw3o

T1 diagnostics: 0.013906404				Harmonic frequencies:					
Cartesian coordinates:				43.72	606.65	1467.21	43.72	606.65	1467.21
C	0.83976200	-0.10296600	0.16782100	99.93	760.3	1480.75	99.93	760.30	1480.75
O	0.33822800	-0.75124000	1.05577600	114.99	817.61	1633.1	114.99	817.61	1633.10
C	2.19551800	-0.43900400	-0.39145400	126.14	962.48	1832.81	126.14	962.48	1832.81
H	2.91062100	0.31747800	-0.06215800	143.27	1003.87	3010.1	144.34	1003.87	3010.10
H	2.51385700	-1.41566500	-0.04028100	162.68	1045.88	3030.04	162.68	1045.88	3030.04
H	2.17502000	-0.41163200	-1.48113700	195.79	1126.4	3067.45	195.79	1126.40	3067.45
C	0.13261300	1.08404900	-0.45335100	255.25	1196.32	3132.71	255.25	1196.32	3132.71
H	0.88095000	1.85482000	-0.67847100	273.97	1214.82	3183.88	273.97	1214.82	3183.88
H	-0.21685200	0.73633500	-1.43504600	405.88	1376.02	3185.66	405.88	1376.02	3185.66
C	-0.99168300	1.61242700	0.35361000	428.21	1405.53	3293.64	428.21	1405.53	3293.64
H	-1.62762100	2.38685800	-0.04458800	473.31	1424.18	3811.78	473.31	1424.18	3811.78
H	-2.74299900	-0.52663300	-0.33162500	498.4	1456.27	3937.07	498.40	1456.27	3937.07
H	-1.11205600	1.30562300	1.38039300				*(With hindered internal rotation analysis)*		
O	-2.12833500	-1.24266400	-0.50664100						
H	-1.51732000	-1.22298200	0.24008100						

pr01 $CH_2 \cdot COCH_2CH_3$

T1 diagnostics: 0.015734770				Harmonic frequencies:					
Cartesian coordinates:				68.47	981.34	1495.7	72.61	981.34	1495.7
C	0.58637200	0.11049000	-0.00000100	218.32	1026.86	1503.1	224.52	1026.86	1503.1
O	0.51361400	1.33180500	-0.00000100	256.29	1082.15	1679.23	256.29	1082.15	1679.23
C	1.86854600	-0.54739100	0.00000400	374.64	1110.99	3052.02	377.92	1110.99	3052.02
H	2.76194200	0.05801400	-0.00000400	410.92	1222.39	3078.44	410.92	1222.39	3078.44
H	1.95033200	-1.62442600	0.00000700	504.91	1279.83	3082.49	504.91	1279.83	3082.49
C	-0.65310400	-0.76452900	-0.00000400	593.29	1388.74	3151.87	593.29	1388.74	3151.87
H	-0.59623500	-1.42355100	-0.87095900	722.29	1419.09	3153.3	722.29	1419.09	3153.3
H	-0.59623300	-1.42356600	0.87093900	789.5	1463.69	3174.61	789.5	1463.69	3174.61
C	-1.94130200	0.03783000	0.00000300	811.58	1472.13	3291.54	811.58	1472.13	3291.54
H	-2.80682100	-0.62244300	-0.00000100				*(With hindered internal rotation analysis)*		
H	-1.99248600	0.68155700	0.87618800						
H	-1.99248800	0.68157200	-0.87617100						

pr02 $CH_3COCH{\cdot}CH_3$

T1 diagnostics: 0.016000900				Harmonic frequencies:					
Cartesian coordinates:				47.5	979.55	1481.82	79.51	979.55	1481.82
C	0.50666300	0.14679700	-0.00008400	89.19	1037.89	1501.26	116.04	1037.89	1501.26
O	0.43177700	1.36979300	-0.00002700	155.19	1050.27	1676.79	176.32	1050.27	1676.79
C	1.84367600	-0.55975400	0.00004900	261.67	1149.97	3042.35	261.67	1149.97	3042.35
H	1.93361900	-1.20035100	-0.87817300	417.32	1227.25	3067.54	417.32	1227.25	3067.54
H	2.64246100	0.17561200	-0.00135400	531.01	1384.81	3086.29	531.01	1384.81	3086.29
H	1.93455200	-1.19778000	0.88005500	607.31	1395.01	3130.66	607.31	1395.01	3130.66
C	-0.67944700	-0.67574500	-0.00013000	670.71	1440.39	3164.36	670.71	1440.39	3164.36
H	-0.55674300	-1.75165700	-0.00019000	803.93	1471	3181.96	803.93	1471	3181.96
C	-2.03963900	-0.09671600	0.00006400	964.74	1479.14	3193.89	964.74	1479.14	3193.89
H	-2.60343500	-0.42993900	0.87552700				*(With hindered internal rotation analysis)*		
H	-1.98810300	0.98902700	-0.00038600						
H	-2.60408800	-0.43074600	-0.87465300						

pr03 $CH_3COCH_2CH_2{\cdot}$

T1 diagnostics: 0.014524166				Harmonic frequencies:					
Cartesian coordinates:				73.02	955.21	1469.24	89.27	955.21	1469.24
C	0.46944300	0.17481000	0.01852100	105.07	1022.59	1481.46	126.08	1022.59	1481.46
O	0.37668300	1.37517900	0.02538300	174.09	1025.92	1850.9	180.10	1025.92	1850.9
C	1.80623700	-0.52238900	-0.04169900	275	1129.04	2988.64	275	1129.04	2988.64
H	1.93435200	-0.95425200	-1.03622400	404	1193.88	3029.04	404	1193.88	3029.04
H	2.60498200	0.18901200	0.14525700	418.18	1201.61	3067.44	418.18	1201.61	3067.44
H	1.84995300	-1.34255500	0.67498600	472.72	1374.45	3132.55	472.72	1374.45	3132.55
C	-0.74886000	-0.73074600	0.06638600	596.97	1400.22	3182.84	596.97	1400.22	3182.84
H	-0.62442300	-1.50804600	-0.69781700	766.8	1430.56	3190.01	766.8	1430.56	3190.01
H	-0.68228000	-1.28221100	1.01647000	800.62	1451.89	3302.56	800.62	1451.89	3302.56
C	-2.03932200	-0.01974100	-0.06465100				*(With hindered internal rotation analysis)*		
H	-2.96335100	-0.57272000	-0.11177900						
H	-2.05768900	1.05774000	-0.06529500						
